# Rapid assessment of feeding intolerance: a systematic approach to reduce time to full enteral feeding in preterm infants

**DOI:** 10.3389/fped.2026.1755920

**Published:** 2026-03-05

**Authors:** Elena Maggiora, Francesco Cresi, Giulia Maiocco, Chiara Peila, Barbara Vania, Elisa Rossi, Danilo A. W. Gavilanes, Diego Gazzolo, Alessandra Coscia

**Affiliations:** 1 Neonatology and Neonatal Intensive Care Unit, Sant'Anna Hospital, Città Della Salute e Della Scienza, Turin, Italy; 2 Department of Public Health and Pediatrics, University of Turin, Turin, Italy; 3 Department of Public Health and Pediatrics, Postgraduate School of Pediatrics, University of Turin, Turin, Italy; 4 Department of Pediatrics and Neonatology, Maastricht University, Maastricht, Netherlands; 5 Neonatal Intensive Care Unit G. D’Annunzio University of Chieti, Chieti, Italy

**Keywords:** enteral nutrition, feeding intolerance, full enteral feeding, intrauterine growth restriction, very preterm infants

## Abstract

**Background:**

Feeding intolerance (FI) is common in very preterm infants and often leads to unnecessary interruptions in enteral nutrition (EN), delaying full enteral feeding (FEF). The absence of standardized criteria contributes to inconsistent management. We evaluated the impact of a structured protocol—Rapid Assessment of Feeding Intolerance (RAFI)—on FEF achievement in preterm infants.

**Methods:**

This single-center, retrospective-prospective superiority cohort study included infants <30 weeks' gestation. Two cohorts were defined: a historical control group (pre-RAFI) and a RAFI group (first implementation phase). The primary outcome was time to FEF (150 mL/kg/day of EN). One-sided statistical tests were used to assess the superiority of RAFI. Stratified analysis was performed for infants with intrauterine growth restriction (IUGR).

**Results:**

Sixty infants were included (30 per group). RAFI infants achieved FEF significantly earlier than controls [median 23.0 (IQR 18.0–30.0) vs. 30.0 (24.0–34.5) days; *p* = 0.041]. Among IUGR infants (*n* = 14), RAFI group achieved FEF earlier [27.00 (24.00–32.00) vs. 35.00 (34.00–61.00) days; *p* = 0.036] at earlier post-menstrual age [33.0 (32.5–34.0) vs. 34.0 (34.0–37.5) weeks; *p* = 0.028] and with a lower weight [1,280 (1,130–1,382) vs. 1,535 (1,325–2,002) g; *p* = 0.048]. A trend towards a shorter duration of central venous catheter (*p* = 0.059) and hospital stay (*p* = 0.064) was observed.

**Conclusions:**

RAFI implementation was associated with earlier achievement of FEF, particularly in IUGR infants. These findings suggest that a structured and standardized approach to feeding intolerance assessment may facilitate nutritional advancement in very preterm neonates.

## 1 Introduction

It is now widely recognized that early enteral nutrition (EN) for very preterm infants plays a crucial role in the development and maturation of the gastrointestinal system, contributes to short- and long-term growth outcomes, and has the potential to reduce inflammation, preventing conditions such as bronchopulmonary dysplasia, retinopathy of prematurity, and supporting brain growth and neurodevelopment (1). While parenteral nutrition is essential in the initial stages following preterm delivery to provide necessary sustenance, it is associated with a range of complications, including central line-associated bloodstream infections, sepsis, central line-associated thrombosis, intrahepatic cholestasis, progressive bone demineralization, depletion of venous access, pneumothorax, chylothorax, pneumomediastinum, and haemorrhage (2, 3). Consequently, achieving full enteral feeding (FEF) as early as possible becomes a critical healthcare goal during the neonatal intensive care unit (NICU) stay.

However, for very preterm infants, the advancement of enteral nutrition can be challenging due to feeding intolerance (FI). It is important to note that despite its prevalence, with more than 50% of infants born weighing less than 2,000 grams being affected by FI (4), there is still no consensus on its definition.

FI can be a benign condition associated with the immaturity of gastrointestinal functions, but it can also signal the impending onset of necrotizing enterocolitis (NEC) (5).

Commonly recognized signs of FI include excessive gastric residual volume (GRV), abdominal distension, and emesis. However, there is a lack of unanimity regarding the definition of these signs and which ones should be considered as warning indicators (6).

Accurate detection of imminent NEC is a crucial aspect of FI diagnosis, as it allows for the application of the most appropriate preventive and therapeutic strategies, including the “nil per os” approach (7).

Furthermore, the absence of standardized protocols for managing FI can lead to interruptions in enteral feeding even when there is no proven risk of NEC, a situation that should be avoided as it delays the achievement of FEF and prolongs infants' exposure to the complications of parenteral nutrition.

In our centre, the RAFI (Rapid Assessment of Feeding Intolerance) protocol was developed as a structured tool to support the clinical assessment and management of feeding intolerance in preterm infants. It was locally developed by a multidisciplinary team according to available literature (8–11) and clinical experience. The protocol aims to guide timely and consistent decision-making, reduce unnecessary feeding interruptions, and facilitate the safe and efficient progression to full enteral feeding.

The aim of this study is to evaluate the effectiveness of the RAFI protocol in promoting the achievement of (FEF) in preterm infants, comparing the historical population (before RAFI implementation) with the new “RAFI-cohort”.

## 2 Material and methods

### 2.1 Study design and population

This is a retrospective-prospective observational cohort study conducted at Sant'Anna Hospital in Turin to evaluate the effectiveness and long-term impact of the RAFI protocol in preterm infants with a gestational age <30 weeks. The RAFI protocol was developed within our center and introduced in January 2019 through structured training and the dissemination of reference materials to all medical and nursing staff.

Two cohorts of preterm infants were included in the analysis:

Controls: infants born between January 2015 and December 2017, before the implementation of RAFI. Each control was matched 1:1 to a RAFI infant using a nearest-available matching approach prioritizing gestational age and selecting the closest match based on birth weight when more than one eligible control was available.
RAFI: infants born between January 2019 and January 2021, after the RAFI implementation.

During the study period, no major changes occurred in the unit's enteral nutrition protocol, including NEC prevention policies, human milk availability, or probiotic use. The introduction of the RAFI protocol represented the only relevant change in clinical practice between the two cohorts and specifically targeted the assessment and management of feeding intolerance, without altering the underlying nutritional approach. The RAFI protocol differs from the previous standard by eliminating routine gastric residual volume (GRV) assessment, standardizing the evaluation of abdominal distension, and incorporating the infant's overall clinical condition into feeding decisions. Feeding progression or interruption is guided by specific signs of feeding intolerance (Table 1), classified into minor and major criteria based on physical examination findings, gastrointestinal symptoms as regurgitation or vomiting, gastric residual features when assessed, or stool patterns, and cardiorespiratory events. The number and persistence of these criteria determine whether feeds are continued, stabilized, reduced, or temporarily interrupted, with reassessment before each meal. A recommended upper limit of 30 mL/kg/day is advised; however, feed advancement was primarily based on overall clinical judgment, including the infant's needs and signs of distress.

**Table 1:** Advancement and interruption of enteral feeding by signs of FI.

Item	Minor criteria	Major criteria
Physical examination	Abdominal distension^a^ grade 1–2	Abdominal distension^a^ grade 3
	Visible bowel ansa	Dyschromic abdominal wall
		Painful abdomen
Regurgitations/vomits	≤2 episodes between 2 feeds or in the previous 3 h (if not fed)	> 2 episodes between 2 feeds or in the previous 3 h (if not fed)
		Bilious vomiting/hematemesis
Gastric residual volumes (GRVs)	GRV < 100% of previous feed (bilious or with hematic fragments)	Hematic/fecaloid GRV
		GRV ≥ 100% of previous feed
Alvus	Mucous stools	Hematic stools
CR events	≥3 CR events^b^/h	≥ 1 severe CR event^c^
0–1 minor criteria	Continue enteral feeding with increments as per protocol (max 30 mL/kg/day)	
2 minor criteria	Stop increasing feeds, re-assess prior to the next feed, and evaluate GRV if not done before	
	If 2 minor criteria in at least 2 consecutive evaluations, consider reducing volume of feed	
1 major criterion or 3 minor criteria	Interrupt enteral feeding and re-assess prior to the next feed	

^a^ Abdominal distension: grade 0 = not distended; grade 1 = distended but not tense; grade 2 = distended/tensed, responsive to gastric suction/rectal stimulation; grade 4 = distended/tensed, not responsive to gastric suction/rectal stimulation.

^b^ Cardiorespiratory (CR) events defined as episodes of apnea lasting more than 5 s if followed by desaturation or bradycardia, episodes of desaturation with blood oxygen saturation <80%, and episodes of bradycardia with heart rate <80 bpm.

^c^ Extreme cardiorespiratory (CR) events defined as CR events requiring resuscitation.

All infants were followed until achievement of full enteral feeding (FEF) or discharge. Relevant clinical outcomes were collected for comparative analysis.

Inclusion criteria were: gestational age <30 weeks, initiation of enteral nutrition within the first 7 days of life, and written informed consent for the use of anonymized clinical data signed by parents.

Exclusion criteria included: requirement for invasive ventilation for more than five consecutive days after birth, major congenital or chromosomal abnormalities, neurological disease, surgical conditions (except abdominal surgery related to prematurity), and confirmed neonatal sepsis.

Clinical data were collected in accordance with the Manual of Operations of the Vermont Oxford Network (12) and extracted from both electronic and paper medical records. Participation in the study and data collection were conducted only after obtaining written informed consent signed by the parents. The study was approved by the Institutional Review Board and the Ethics Committee of the AOU Città della Salute e della Scienza di Torino (ID:0043331/2018) and was conducted in accordance with the Declaration of Helsinki.

### 2.2 Outcomes

The primary outcome of the study was the time required to reach full enteral feeding (FEF), defined as the achievement of an enteral intake of 150 mL/kg/day. This endpoint was selected as a clinically relevant indicator of feeding tolerance and gastrointestinal adaptation in preterm infants and was measured in days from the initiation of enteral nutrition.

Secondary outcomes included feeding and growth parameters, and clinical morbidities associated with feeding intolerance and prematurity.

Feeding-related outcomes were:

Interruptions of enteral nutrition (EN): the number of clinician-initiated interruptions of enteral feeding per day, counted independently of how many scheduled feeds were missed during each interruption.
Not given feeds: the total number of scheduled feeds not administered per day due to any interruption of EN.

Clinical outcomes included:

Incidence of necrotizing enterocolitis (NEC), diagnosed according to the modified Bell's staging criteria (13).
Episodes of sepsis.
Duration of central venous catheter (CVC) use (in days).
Length of hospital stay (in days).

### 2.3 Statistical analysis

In this superiority study, continuous variables were expressed as mean ± standard deviation or as median and interquartile range, depending on data distribution. Comparisons between the two cohorts (Controls and RAFI) were performed using one-sided Mann–Whitney U tests or unpaired *t*-tests, depending on normality assumptions, with the alternative hypothesis that RAFI was superior in reducing the outcome of interest. Categorical variables were compared using one-sided Fisher's exact test, assuming a lower incidence in the RAFI group. NEC, sepsis, and PDA were treated as safety outcomes and were analyzed using two-sided tests. A *p*-value < 0.05 was considered statistically significant.

A stratified analysis was also conducted on the subgroup of infants with intrauterine growth restriction (IUGR), who are known to present specific characteristics related to feeding tolerance and nutritional adaptation.

All statistical analyses were performed using Stata Statistical Software: Release 18 (StataCorp LLC, College Station, TX, USA).

### 2.4 Sample size

Sample size estimation was based on an unpaired one-sided *t*-test for independent groups, consistent with the superiority design of the study. Using unpublished data from our centre, the mean time to achieve full enteral feeding (FEF) in the control population was estimated at 30 days with a standard deviation of 9 days. Assuming a clinically meaningful difference of 7 days in favour of the RAFI group, with a one-sided alpha of 0.05 and a power of 80%, the required sample size was calculated to be 26 infants per group. To account for potential data loss and ensure adequate statistical power, we decided to enrol 30 infants per group.

## 3 Results

A total of 60 preterm infants with gestational age <30 weeks were included in the present analysis, evenly divided between the Control and RAFI groups. The median gestational age at birth was 28.0 weeks (IQR: 26.6–29.0), and the median birth weight was 990 grams (IQR: 790–1,225). The proportion of infants classified as intrauterine growth restricted (IUGR) was 27%. Detailed baseline characteristics for each group are presented in Table 2.

**Table 2:** Clinical and anthropometric characteristics at birth of the study population (*n* = 60).

Variable	Control (30)	RAFI (30)
Weeks of GA, *n*	28 (27–28)	28 (27; 29)
Female, *n* (%)	16 (53)	15 (50)
Weight, g	990 (850; 1,125)	1,035 (885; 1,157)
Weight, SD (24)	0.14 (−0.69; 0.62)	0.14 (−0.32; 0.43)
Length, cm	35.00 (34.35; 36.10)	36.30 (34.45; 38.35)
Head circumference, cm	24.90 (24.50; 26.20)	25.90 (24.62; 26.70)
IUGR, *n* (%)	7/30 (23)	7/30 (23)
RDS prophylaxis, *n* (%)	4/29 (14)	2/30 (7)
Surfactant, *n* (%)	16/30 (53)	16/30 (53)
Need for resuscitation, *n* (%)	30/30 (100)	28/30 (93)

### 3.1 Primary outcome

**Figure 1:**
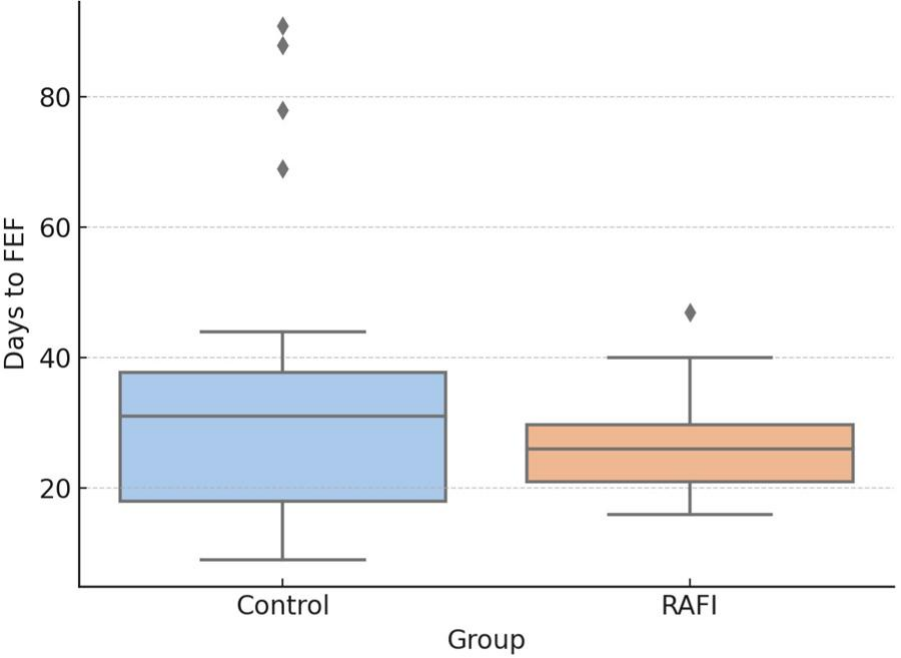
Time to reach full enteral feeding (FEF), defined as an enteral intake of 150 mL/kg/day.

The primary outcome of the study was the time to reach full enteral feeding (FEF), defined as an enteral intake of 150 mL/kg/day. In the overall population, infants in the RAFI group reached FEF significantly earlier than those in the historical control group [median (IQR): 26.00 (21.00–29.75) vs. 31.00 [18.00–37.75] days; *p* = 0.041], supporting the effectiveness of the protocol in promoting earlier feeding progression (Figure 1).

**Figure 2:**
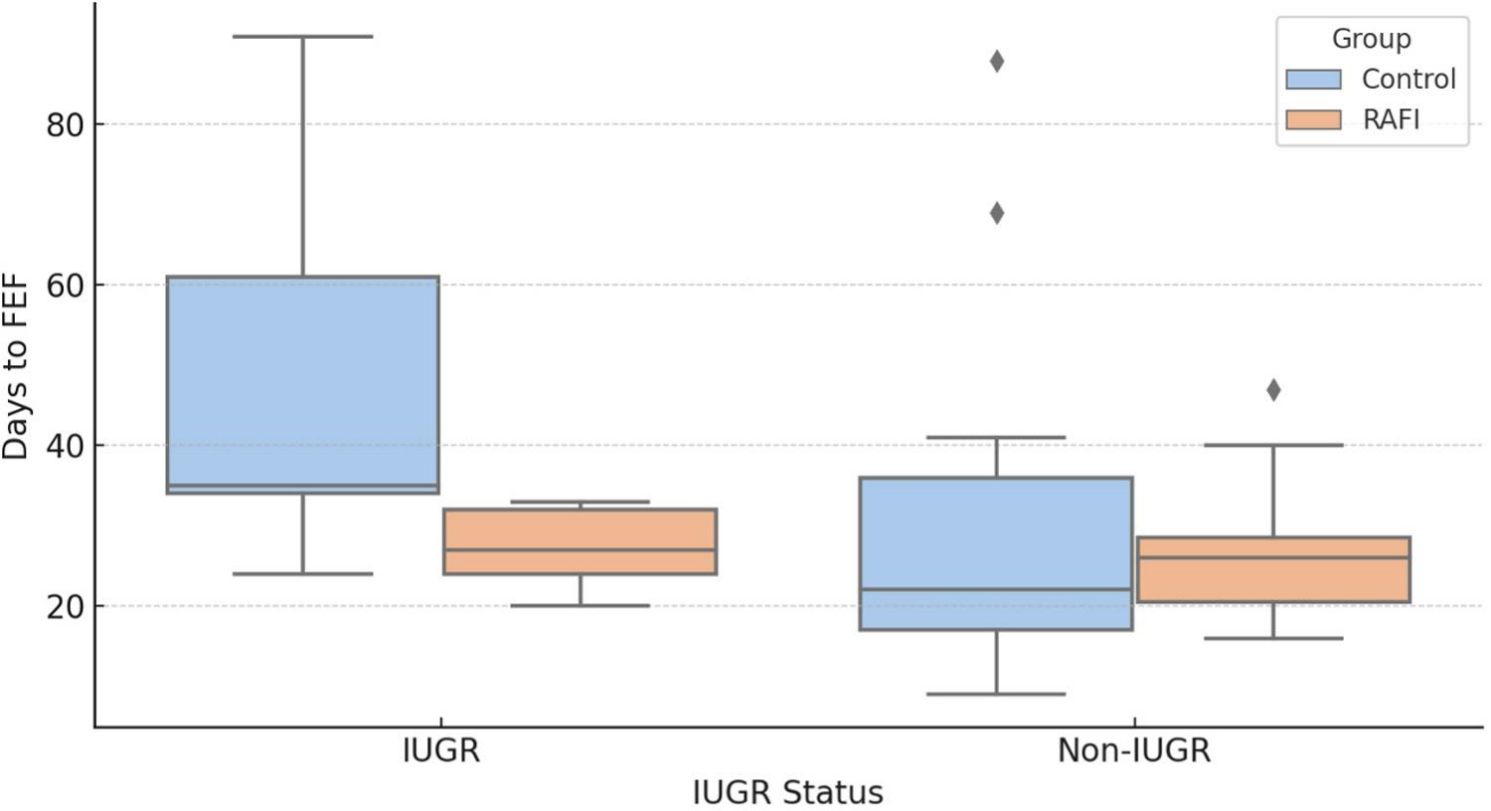
Time to reach full enteral feeding (FEF) in IUGR and non-IUGR infants.

To further explore whether this effect was consistent across different clinical subgroups, we performed a stratified analysis based on intrauterine growth restriction (IUGR). Among IUGR infants, those exposed to RAFI reached FEF earlier compared to controls [27.00 (24.00–32.00) vs. 35.00 (34.00–61.00) days; *p* = 0.036], indicating a substantial benefit of the protocol in this particularly vulnerable population. In contrast, no statistically significant difference was found among non-IUGR infants [26.00 (20.50–28.50) vs. 22.00 (17.00–36.00) days; *p* = 0.223], suggesting that the effect of RAFI may be more pronounced in infants with impaired intrauterine growth (Figure 2).

### 3.2 Secondary outcomes

**Figure 3:**
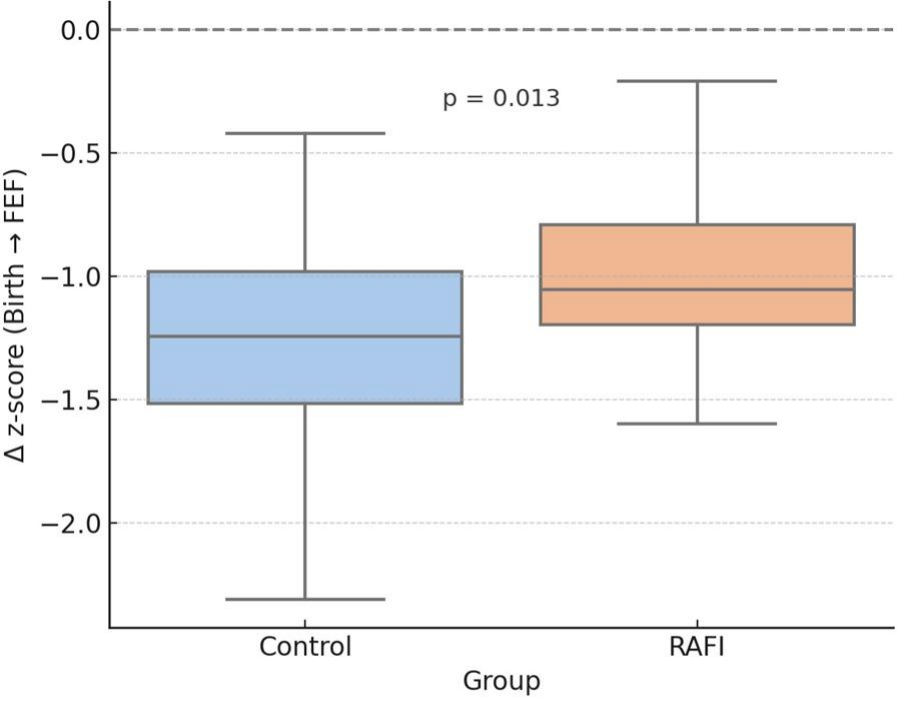
Weight Δz-score from birth to the achievement of FEF (24).

Among the secondary outcomes, significant differences were observed in the duration of CVC use and the number of enteral nutrition interruptions per day between the control and RAFI groups. Full details of the secondary outcomes analysis are reported in Table 3. The RAFI group showed a reduced decline in weight z-score from birth to the achievement of FEF compared to the control group [−1.05 (−1.20 to −0.79) vs. −1.24 (−1.52 to −0.98) SD; *p* = 0.013]. This trend is illustrated in Figure 3.

**Table 3:** Primary and secondary outcomes of the study population (*n* = 60).

Variable	Control (30)	RAFI (30)	*p*
Primary outcome			
Days at FEF, *n*	31.00 (18.00; 37.75)	26.00 (21.00; 29.75)	0.041*
Secondary outcomes			
PMA at FEF, weeks	31.50 (30.25; 33.75)	32.00 (31.00; 33.00)	0.148
Weight at FEF, g	1,335 (1,115; 1,552)	1,317 (1,201; 1,473)	0.269
Weight at FEF, SD (24)	−1.31 (−1.75; −0.65)	−1.04 (−1.41; −0.71)	0.879
Length at FEF, cm	38.35 (36.95; 40.42)	38 (36.83; 40.45)	0.213
Head circumference at FEF, cm	27.50 (26.73; 28.35)	27.25 (26.57; 28.50)	0.641
Interruptions of EN, *n*	4.00 (2.00; 8.75)	3.00 (1.25; 5.00)	0.021*
Not given feeds, *n*	7.00 (2.25; 18.50)	7.00 (1.25; 17.25)	0.043*
Days of CVC, *n*	30.00 (20.00; 36.75)	30.50 (26.00; 37.50)	0.321
NEC, *n* (%)	0/30 (0)	1/30 (3)	1.000
Sepsis, *n* (%)	2/30 (7)	2/30 (7)	1.000
PDA, *n* (%)	6/30 (20)	12/30 (40)	0.929
Days of hospitalization, days	60 (51; 64)	61 (47; 69)	0.485
Weight at discharge, g	2,035 (1,862; 2,277)	2,300 (1,860; 2,560)	0.767
Weight at discharge, SD (24)	−1.48 (−2.04; −0.99)	−1.28 (−1.87; −1)	0.682
Lenght at discharge, cm	43.10 (41.25; 44.72)	44.10 (41.98; 45.65)	0.756
Head circumference at discharge, cm	31.00 (30; 32.50)	32.10 (30.65; 33.28)	0.853

* *p* < 0.05.

In Table 4 are reported the secondary outcomes analysis of the IUGR population.

**Table 4:** Primary and secondary outcomes of the IUGR population (*n* = 14).

Variable	Control (7)	RAFI (7)	*p*
Primary outcome			
Days at FEF, *n*	35.00 (34.00; 61.00)	27.00 (24.00; 32.00)	0.036*
Secondary outcomes			
PMA at FEF, weeks	34.00 (34.00; 37.50)	33.00 (32.50; 34.00)	0.028*
Weight at FEF, g	1,535 (1,325; 2,002)	1,280 (1,130; 1,382)	0.048*
Weight at FEF, SD (24)	−2.39 (−2.50; −2.12)	−1.97 (−2.19; −1.76)	0.894
Length at FEF, cm	37.50 (37.10; 39.30)	36.00 (35.65; 37.00)	0.095
Head circumference at FEF, cm	27.50 (27.50; 28.80)	27.00 (26.75; 27.55)	0.171
Interruptions of EN, *n*	4.00 (1.50; 13.00)	5.00 (4.00; 5.00)	0.194
Not given feeds, *n*	6.00 (3.00; 46.00)	20.00 (13.00; 21.00)	0.237
Days of CVC, *n*	35.00 (32.00; 60.50)	30.00 (26.50; 32.50)	0.059
NEC, *n* (%)	0/7 (0)	0/7 (0)	1.000
Sepsis, *n* (%)	1/7 (14)	0/7 (0)	1.000
PDA, *n* (%)	0/7 (0)	1/7 (14)	0.735
Days of hospitalization, days	64.00 (55.00; 77.00)	54.00 (49.00; 61.50)	0.064
Weight at discharge, g	1,840 (1,747; 2,182)	1,850 (1,780; 1,992)	0.176
Weight at discharge, SD (24)	−2.39 (−2.88; −2.15)	−2.59 (−2.85; −2.29)	0.554
Lenght at discharge, cm	41.90 (40.65; 43.75)	41.75 (40.38; 42.22)	0.141
Head circumference at discharge, cm	30.00 (29.80; 31.90)	30.90 (30.18; 31.18)	0.227

* *p* < 0.05.

## 4 Discussion

In this study, the implementation of the RAFI protocol for the management of feeding intolerance (FI) in very preterm infants was associated with earlier achievement of full enteral feeding (FEF), fewer interruptions of enteral nutrition, and improved short-term growth outcomes. These effects were especially pronounced among infants with intrauterine growth restriction (IUGR), a population typically at higher risk for FI-related complications. Compared to their matched controls, IUGR infants managed with RAFI reached FEF an average of 8 days earlier and were discharged 10 days sooner. Although not all differences reached statistical significance—likely due to limited sample size—the trends consistently favored the RAFI group, supporting its potential clinical benefit.

Diagnosis of FI remains one of the main causes of disruption to feeding plans in very preterm infants, primarily due to the fear of evolving necrotizing enterocolitis (NEC). A major limitation in current practice is the absence of a standardized and widely accepted definition of FI. Clinicians often rely on a nonspecific set of signs and symptoms with various interpretations. When these are mistakenly attributed to pathological FI, they may lead to unjustified feed interruptions in infants without true gastrointestinal pathology. Thus, the morbidity associated with FI often stems from the inability to distinguish benign, developmentally appropriate signs from early NEC indicators (14).

A recent systematic review by Weeks et al. (15) confirmed the lack of consensus on FI definitions and revealed considerable heterogeneity in clinical practice. Criteria such as abdominal distension, blood in stool, vomiting, bilious aspirates, and gastric residual volume (GRV) are commonly cited but combined in numerous ways across studies, with minimal consistency in thresholds and timing. Importantly, several definitions include signs more typically associated with NEC, potentially contributing to clinical confusion. While bowel rest remains essential for NEC management (16), the approach to suspected FI in otherwise stable infants should favor cautious feed reduction and close reassessment over total fasting (17).

Within this context, the RAFI protocol provides a structured and clinically pragmatic approach to FI assessment, which can promote more consistent and appropriate feeding decisions. In our study, RAFI implementation led to earlier achievement of FEF—the study's primary outcome—and reduced the number of feed interruptions and not given feeds. Earlier discontinuation of parenteral nutrition also has implications for patient safety and resource use.

Interestingly, the earlier achievement of FEF in the RAFI group did not translate into a significantly shorter duration of central venous catheter (CVC) use. This may reflect a cautious clinical approach: neonatologists might have hesitated to remove the CVC in infants who, although nutritionally autonomous, still had a relatively low weight at the time of FEF achievement. This highlights a potentially modifiable practice pattern and suggests that future iterations of feeding protocols may benefit from incorporating weight-based or condition-based guidance for CVC removal decisions.

The benefits of RAFI were particularly evident in IUGR infants, who are especially vulnerable to FI and are often managed more conservatively due to their fragility (18). In this subgroup, protocol-guided management led to meaningful improvements in feeding progression and earlier discharge. Although the reduction in NICU stay in the overall cohort did not reach statistical significance, the observed trend remains clinically relevant. However, secondary outcomes were not adjusted for multiple comparisons and should be considered exploratory. The IUGR subgroup analysis, based on a very small sample size, is hypothesis-generating and requires confirmation in larger studies.

Other FI management strategies have been proposed, including a recent algorithm by Nesterenko et al. (19), introduced as part of a broader NEC prevention initiative. Their approach focused primarily on GRV volume and color, which are known to be unreliable in extremely preterm infants. GRVs up to 3 mL and greenish aspirates may be physiological in this population (20), and GRV volume may vary based on feeding position, tube location, and aspiration technique (21). As such, GRV alone may not accurately reflect pathological FI and could unnecessarily delay feeding progression (22).

Rapid progression to FEF is crucial for promoting adequate growth (23). In our study, infants managed under the RAFI protocol exhibited less decline in weight z-score from birth to FEF compared to controls, particularly in the non-IUGR subgroup. Although absolute weight at FEF was slightly lower in RAFI infants—due to earlier achievement of milestones—z-score trajectories reflected better growth preservation. These findings support the utility of RAFI in facilitating both feeding advancement and nutritional adequacy.

To our knowledge, RAFI represents one of the first structured and pragmatic feeding intolerance management protocols to integrate multiple clinical domains—including physical examination findings, gastrointestinal symptoms, cardiorespiratory events, and selective use of gastric residuals—into a standardized decision-making framework. Our results suggest both its efficacy and safety.

In the absence of universally accepted protocols for feeding intolerance assessment our results are consistent with previous studies (14, 15) showing that structured, protocol-driven approaches, despite methodological heterogeneity, are associated with earlier advancement of enteral nutrition in preterm infants.

Nevertheless, several limitations must be acknowledged. This was a single-center study with a relatively small sample size and retrospective enrollment of the control group. The use of a historical control group represents a potential source of temporal bias. Even though unmeasured confounding cannot be entirely excluded, no substantial changes in NEC prevention strategies or nutritional policies occurred in our unit during the study period. The RAFI protocol was the only relevant modification introduced, supporting the interpretation that the observed differences are primarily related to standardized feeding intolerance assessment rather than broader temporal trends.

In addition, we excluded infants requiring invasive mechanical ventilation for more than 5 days to focus on a population of stable infants in whom enteral feeding could be safely implemented, while those infants in our unit who require prolonged ventilation are usually managed with a more individualized, patient-specific approach. This criterion may limit the generalizability of our results, particularly for extremely preterm infants (<28 weeks), who are more likely to require prolonged respiratory support.

It is interesting to note that a higher prevalence of patent ductus arteriosus was observed in the RAFI group. As PDA may be associated with reduced feeding tolerance and a more cautious approach to enteral feeding advancement, this imbalance may have partially influenced feeding-related outcomes and potentially attenuated the observed beneficial effect of the RAFI protocol.

Although the matched design strengthens the validity of our comparisons and suggests potential benefits of structured protocol-guided management, causal inferences cannot be made due to the retrospective observational design. In particular, safety outcomes such as NEC were not powered to detect differences, and no firm conclusions regarding the impact of RAFI on rare but severe adverse events can be drawn. A larger, multicenter prospective study would therefore be needed to confirm these findings and more robustly assess both efficacy and safety.

A randomized controlled trial would be difficult to implement due to the inability to blind clinical decisions and ethical concerns regarding different management approaches in similar patients.

## 5 Conclusion

The RAFI protocol appears to be a safe and effective strategy for the management of feeding intolerance in very preterm infants. Its implementation was associated with a significantly earlier achievement of full enteral feeding compared with standard care. This effect was particularly evident in IUGR infants, who reached full enteral feeding at an earlier post-menstrual age and showed a more favorable weight profile. Further multicenter studies are needed to confirm these findings and to better define the impact of the RAFI protocol on broader clinical outcomes.

## Data Availability

The raw data supporting the conclusions of this article will be made available by the authors, without undue reservation.
